# Neural competition via lateral inhibition between decision processes and not a STOP signal accounts for the antisaccade performance in healthy and schizophrenia subjects

**DOI:** 10.3389/fnins.2015.00005

**Published:** 2015-01-29

**Authors:** Vassilis Cutsuridis

**Affiliations:** Institute of Molecular Biology and Biotechnology, Foundation for Research and Technology – HellasHeraklion, Greece

**Keywords:** decision making, neural network model, eye movement, accumulator, superior colliculus, cortex

Decision making is the process of accumulating evidence about the world and the utility of possible outcomes (Cutsuridis, 2010). A paradigm often used by behavioral neuroscientists to investigate decision processes is the antisaccade paradigm (see Figure 1A; Hallett, 1978). In the antisaccade paradigm subjects are required to suppress an erroneous saccade (error prosaccade) toward a peripheral stimulus and instead make an eye movement to a position in the opposite hemifield (antisaccade). The response repertoire of a subject performing the antisaccade task has been reported to be: (1) the subject makes an erroneous response (i.e., looking toward the peripheral stimulus), (2) the subject makes the antisaccade (i.e., looking in the opposite direction of the peripheral stimulus, and (3) the subject makes an erroneous response followed by a corrected antisaccade (Evdokimidis et al., 2002).

**Figure 1:**
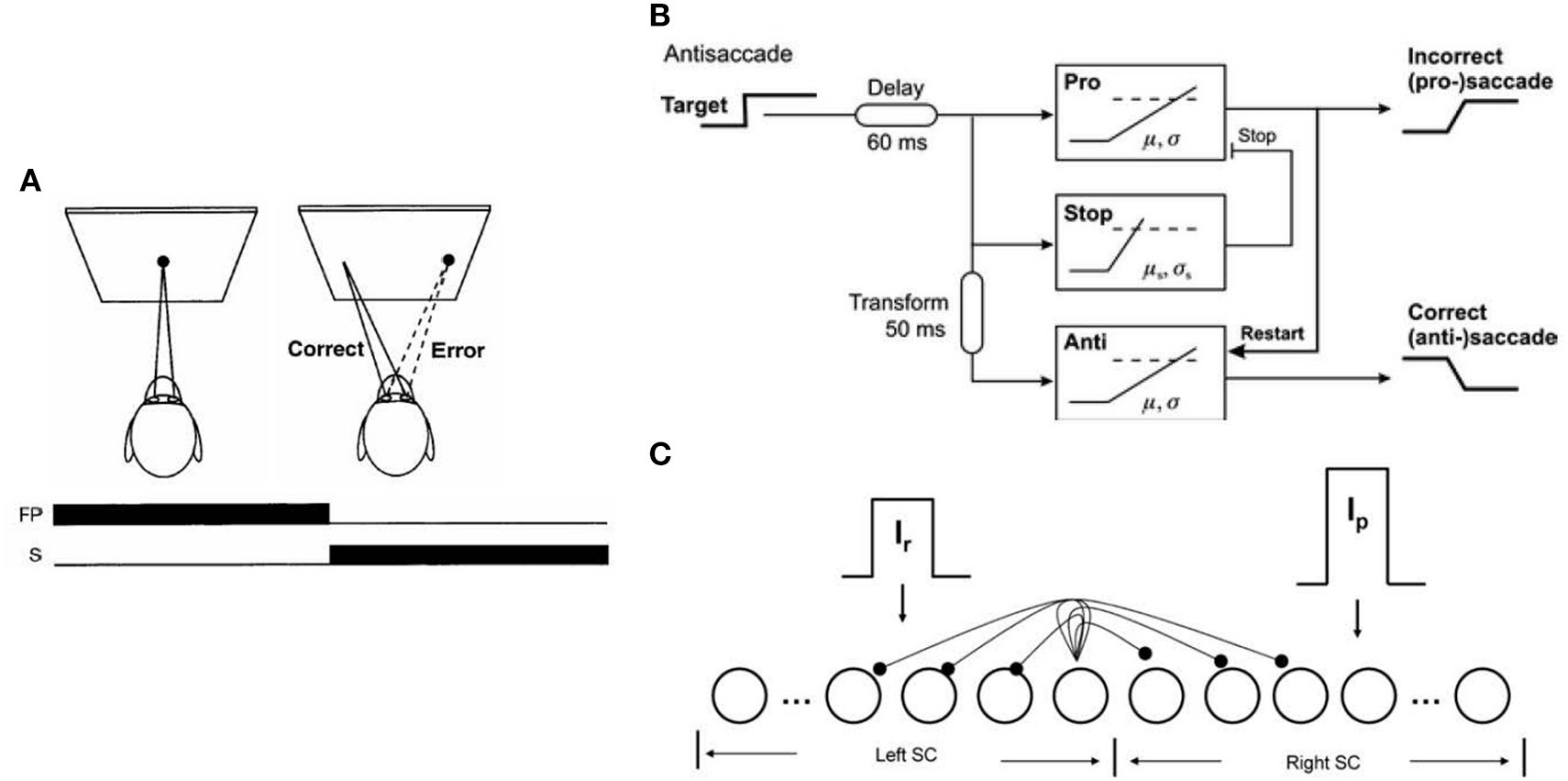
**(A)** Mirror antisaccade task. **(B)** Noorani and Carpenter (2014) model for antisaccades (reprinted with permission from Noorani and Carpenter, 2014). **(C)** Cutsuridis et al. (2014) neural network model of the superior colliculus (SC) for antisaccades in healthy and schizophrenia subjects (reprinted with permission from Cutsuridis et al., 2014). Neurons are represented as nodes. Short-range lateral excitation and long distance lateral inhibition was assumed between all nodes in the network. The left half of the network represented the left SC, whereas the right half represented the right SC. The left SC was activated by a reactive input *Ir* (error prosaccade decision signal), whereas the right SC was activated by a planned input *Ip* (antisaccade decision signal). The strengths of the inputs were not equal.

Many computer models of decision making have been advanced. In these models, decision making involves a gradual accumulation of evidence with a variable rate *r*. When this accumulation process crosses a threshold *S*_*T*_, then a response is generated. Response time (RT) is then the time from the onset of the decision process till when the decision processes crosses *S*_*T*_.

Recently the LATER (Linear Approach to Threshold at Ergodic Rate) model has been extended in the realm of the antisaccade task (see Figure 1B): (Noorani and Carpenter, 2014). The model consisted of three accumulator units racing to threshold: an “anti” unit, a “pro” unit, and a “stop” unit. The “stop” unit prevented the “pro” unit from reaching threshold, thus allowing the “anti” unit to reach a different threshold a little later. The authors hypothesized that the threshold level of the “pro” unit was higher than the “anti” unit's threshold, reflecting this way the advice given by the experimenters to every subject to avoid errors. How often the “stop” unit canceled the “pro” unit depended on its rate of accumulation (μ) and its variance (σ^2^). In the case the “pro” unit reached the threshold first, it restarted the “anti” unit allowing it to reach the threshold and generate the antisaccade response. The model's performance was contrasted against the performance of five healthy subjects performing the antisaccade task. The model captured *most* of the response repertoire observed in the antisaccade task, namely the antisaccades and error prosaccades followed by corrected antisaccades, but *not* the error prosaccades, their corresponding latency distributions and the error response rate. Despite the model's successes, the model had several shortcomings.

First, the model is unable to produce *just* the error prosaccade. This shortcoming is inherent in the model. The authors postulated that if the STOP signal did not prevent the error prosaccade response, then the “pro” unit will *always* restart the “anti” unit (Noorani and Carpenter, 2014). This means the error prosaccades followed by corrected antisaccades will always be produced. If the “stop” unit did prevent the “pro” unit, then the “anti” unit would not re-start, and an antisaccade response would be generated (Noorani and Carpenter, 2014). In either scenario, *just* an error prosaccade response cannot be generated. Psychophysical studies of the antisaccade task (Evdokimidis et al., 2002) have reported that subjects make *just* erroneous prosaccades, but their response frequency is low.

Furthermore, the model implies that the latency of the corrected antisaccade is the result of the linear sum of latencies of the error prosaccade and the antisaccade minus the latency of the STOP activity. This shortcoming is also inherent in the model, because its units are considered linear encoders of the input information. In contrast, all neurons in the brain (units in the model) non-linearly transform the sum of the dendritic outputs before they generate a neuronal response. Dendritic subunits are perhaps the only linear encoders of incoming information (Polsky et al., 2004).

Moreover, the model postulates the existence of a STOP signal, which occasionally stops the error prosaccade response and indirectly allows just the antisaccade response to be expressed (Noorani and Carpenter, 2014). Many past experimental studies have speculated the origins of such a signal (basal ganglia, FEF, DLPFC, etc.) (Munoz and Everling, 2004), but recent experimental evidence has challenged the existence of such a signal (Everling and Johnston, 2013). On the other hand, computational studies have suggested that such a STOP signal might operate as a top-down excitatory signal which activates the local inhibitory neurons in a distant area, which in turn inhibit their neighboring excitatory neurons (Brown et al., 2004).

Attractive alternatives of the Noorani and Carpenter (2013, 2014) models of the antisaccade performance are the models of Cutsuridis et al. (2007, 2014) (see Figure 1C). Cutsuridis et al. (2007) proposed that competition via lateral inhibition (Takahashi et al., 2005; Phongphanphanee et al., 2014) between neurons encoding the volitional antisaccade and neurons encoding the erroneous prosaccade is sufficient to accurately reproduce the error rate and antisaccade, error prosaccade and corrected antisaccade latency distributions of antisaccade data from a large cohort of healthy subjects (Evdokimidis et al., 2002). The model's neurons were non-linear accumulators of incoming information and represented the build-up neurons experimentally recorded in the superior colliculus (SC) (Munoz and Wurtz, 1995).

Recently, Cutsuridis et al. (2014) extended their model in the realm of schizophrenia. Their model showed in a quantitative way why the antisaccade performance of schizophrenia patients is so poor. It predicted that this performance is not due to a deficit in the top-down inhibitory control of the erroneous response as many speculated, but instead it is a product of the competition between the neuronal representations of the erroneous prosaccade and antisaccade responses in the superior colliculus. The model was successful at capturing the response repertoire (error rates, the median antisaccade, median error prosaccade and median corrected antisaccade latencies as well as the antisaccade, error prosaccade and corrected antisaccade distributions) of both healthy and schizophrenia subjects [see Figures 1, 3 and 4 in Cutsuridis et al. (2014) study].

Overall, competition via lateral inhibition between non-linear accumulator neurons seems to be a better mechanism than the “stop-and-restart” mechanism of Noorani and Carpenter (2014), because it captures the *full* antisaccade performance (latencies of error prosaccades, antisaccades and corrected antisaccades and error rates) of healthy and diseased subjects performing the antisaccade task. Other parameters that may affect the antisaccade performance are the differential strengths of the erroneous prosaccade and the volitional antisaccade signals, or different baseline and/or threshold levels. Although recent experimental evidence has just demonstrated that lateral interactions within SC intermediate segment are more suitable for faithfully accumulating subthreshold signals for saccadic decision-making (Phongphanphanee et al., 2014), a lot more work needs to be done to conclusively show that a STOP-and-restart mechanism is unnecessary in the decision making process. An experimental study in that direction was recently published by Everling and colleagues and challenges the (prevailing) idea of a suppressive/inhibitory influence (STOP signal in the Noorani and Carpenter model) of prefrontal cortical areas on reflexive, erroneous prosaccade generation in this paradigm (Everling and Johnston, 2013 for a recent review).

## Conflict of interest statement

The author declares that the research was conducted in the absence of any commercial or financial relationships that could be construed as a potential conflict of interest.
